# Robust and accurate corneal interfaces segmentation in 2D and 3D OCT images

**DOI:** 10.3389/fmed.2024.1381758

**Published:** 2024-03-18

**Authors:** Xueli Zhu, Wei Huang, Shaodong Ma, Quanyong Yi

**Affiliations:** ^1^Department of Ultrasound, The First Affiliated Hospital of Ningbo University, Ningbo, China; ^2^Institute of Biomedical Engineering, Ningbo Institute of Materials Technology and Engineering, Chinese Academy of Sciences, Ningbo, China; ^3^Department of Biomedical Engineering, Hainan University, Hainan, Haikou, China; ^4^Ningbo Eye Hospital, Wenzhou Medical University, Ningbo, China

**Keywords:** segmentation, optical coherence tomography, cornea, layer, eye

## Abstract

Segmentation of corneal layer interfaces in optical coherence tomography (OCT) images is important for diagnostic and surgical purposes, while manual segmentation is a time-consuming and tedious process. This paper presents a novel technique for the automatic segmentation of corneal layer interfaces using customized initial layer estimation and a gradient-based segmentation method. The proposed method was also extended to three-dimensional OCT images. Validation was performed on two corneal datasets, one with 37 B-scan images of healthy human eyes and the other with a 3D volume scan of a porcine eye. The approach showed robustness in extracting different layer boundaries in the low-SNR region with lower computational cost but higher accuracy compared to existing techniques. It achieved segmentation errors below 2.1 pixels for both the anterior and posterior layer boundaries in terms of mean unsigned surface positioning error for the first dataset and 2.6 pixels (5.2 μ*m*) for segmenting all three layers that can be resolved in the second dataset. On average, it takes 0.7 and 0.4 seconds to process a cross-sectional B-scan image for datasets one and two, respectively. Our comparative study also showed that it outperforms state-of-the-art methods for quantifying layer interfaces in terms of accuracy and time efficiency.

## 1 Introduction

Optical coherence tomography (OCT) can produce detailed cross-sectional images of internal structures in biological tissues (1). Because of its non-invasive and non-contact characteristics, it has been widely used in clinical ophthalmology (1–3), particularly in the retina (1, 2) and cornea (3). Measurements derived from OCT images, such as corneal layer thickness and curvature, can provide important diagnostic information for the management of ectasia, angle assessment, corneal abnormalities and anterior segment tumors (4). Reliable and accurate segmentation methods are required for automatic processing of corneal OCT images to obtain corneal parameters (5, 6), while manual segmentation is not feasible due to the large volume of OCT data generated in clinics.

Several approaches to automated corneal segmentation have been proposed to address the aforementioned issue, with varying degrees of success. Li et al. (5, 7) proposed a fast active contour (FAC) algorithm with second-order polynomial fitting for automated corneal segmentation. Eichel et al. (8) presented a semi-automatic segmentation method using enhanced intelligent scissors and a global optimization method. Shen et al. (9) used a novel method for the anterior segment without segmenting the posterior surface. However, none of the above methods can effectively deal with image regions with a low signal-to-noise ratio (SNR) or artifacts introduced during image acquisition, such as the central and horizontal artifacts described in Section 2. More robust methods have been proposed in recent years. LaRocca et al. (10) presented an approach based on graph theory and dynamic programming with better segmentation performance in terms of robustness against artifacts. A customized Hough transform and refinement using Kalman filtering by Zhang et al. (11) is proposed for low computational cost. However, these studies modeled the interface as a parabola, which is not suitable for uneven layer interfaces. Furthermore, extrapolation into the low SNR region is an inaccurate way to segment corneal boundaries. William et al. proposed a level set with shape constraint model (12) and a graph cut model (13), but both require customized optimal weighting. Deep learning based methods have emerged in recent years. dos Santos et al. (14) proposed a modified U-net model with fewer parameters and fast processing speed. Unfortunately, deep learning techniques require a large amount of labeled data and intensive computational effort for training.

To address the above-mentioned limitations, we propose a novel gradient-based segmentation technique for corneal layer boundaries in this paper. It not only works in two-dimensional (2D) B-scans, but also can be extended to three-dimensional (3D) corneal images segmentation. The proposed method is able to detect the corneal layer boundaries accurately with lower computational cost compared to other state-of-the-art methods, by equipping with novel initial estimation and refinement techniques. The proposed method has been evaluated on two newly-constructed AS-OCT datasets with expert manual annotation, and the results have demonstrated the superiority.

## 2 Materials and methods

**Figure 1:**
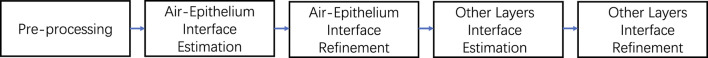
Flowchart of the proposed method to segment the corneal layer interface.

The proposed method involves three key stages: pre-processing, estimation and refinement of the anterior corneal surface, and estimation and refinement of the other layers including the posterior surface and the epithelial-stromal interface (if visible). The whole process is illustrated by the flowchart in Figure 1. In this paper, the following notations are used to describe the proposed segmentation technique: *Y* and *X* denote the depth and width of an image, respectively. The width and height of an image range from 1 to *X* and from 1 to *Y* respectively. The intensity of a pixel at (*x, y*) of an image *I* is represented by *I*(*x, y*).

### 2.1 Materials

This study was approved by the ethics committee of the Cixi Institute of Biomedical Engineering, Chinese Academy of Sciences, and adhered to the principles of the Declaration of Helsinki. Written informed consent was obtained from each subject before they participated in the study.

Two datasets were used in the experiments. Dataset1 consists of 37 anterior segment OCT (AS-OCT) B-scan images of healthy eyes acquired with a Visante AS-OCT system [see Williams et al. (13) for details]. Briefly, each image covers a 16 *mm* wide region sampled by 256 A-scans of 1024 points to a depth of 8 *mm*. The pixel resolution is therefore 60μ*m*×18μ*m*. All images were manually delineated by two ophthalmologists, one of whom marked the images twice in a masked fashion. All 37 images were used as test data for validation.

Dataset 2 is a 3D volume scan of a porcine cornea acquired with an in-house spectral domain OCT device at a scan rate of 100 μ*s* per A-scan using a light source with a central wavelength of 840 *nm*. It consists of 421 raster B-scan images of a 15.1 *mm* region. The images have an axial resolution of 1.9 μ*m* and a transverse resolution of 15 μ*m* with a gap of 20 μ*m* between consecutive B-scans. Three visible layer interfaces (air-epithelium, epithelium-stroma and endothelium-aqueous) of randomly selected 6 B-scans were manually marked twice by an experienced grader for validation purposes.

All images in dataset 1 generated by AS-OCT were used to evaluate the performance of the algorithm. These images were acquired using the Visante AS-OCT system, which is a time-domain system that acquires images at 2,000 axial scans per second in 1,300 nm infrared light. Each B-scan, 37 B-scans in total, consists of 816 A-scans and 406 lateral pixels in each A-scan. The scan width and depth are 16 *mm* and 8 *mm* respectively. The pixel resolution is therefore 19.70 μ*m* × 19.60 μ*m*. The layer interfaces for all 37 test datasets were marked by three different graders simultaneously. Test data for the second dataset, obtained from a home-made OCT, was generated by randomly selecting 6 B-scans from a pool of 421 OCT images. The layer boundaries for the test data were then delineated by an experienced grader. The scan rate of the home-built spectral domain OCT system is 100 *ms* in each A-scan using a light source with a central wavelength of 840 *nm*. Each B-scan image contains 1000 A-scans of 15.11 *mm* with 1024 pixels of 1.9511 *mm*. The pixel resolution for the second dataset is therefore 1.905 μ*m* × 15.113 μ*m*.

### 2.2 Pre-processing

**Figure 2:**
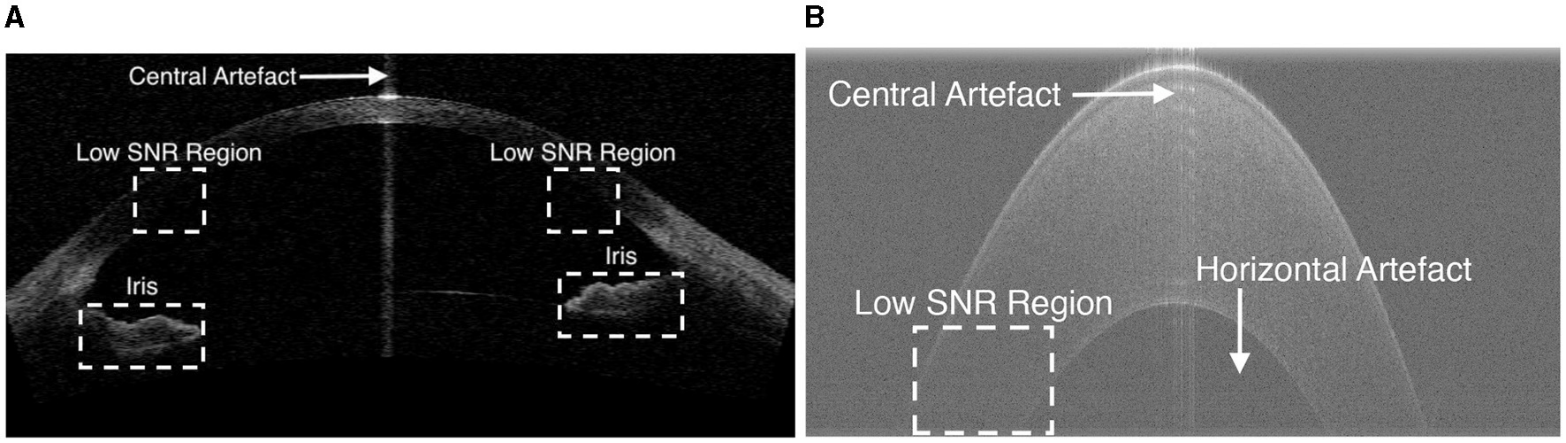
Example OCT images showing low SNR regions, horizontal artifacts, central artifacts and the iris. **(A)** An example image in Dataset1. **(B)** An zoomed-in cornea image in Dataset2.

The first step is to remove unwanted structures and noise (e.g., the iris and high intensity artifacts at the apex position) from the image, as shown in Figure 2, to reduce their detrimental effect on segmentation performance. This is achieved by cropping the image, after which all the content remaining in the image becomes a region of interest (ROI). It is observed that the apex of the cornea in OCT images has a relatively higher intensity than the region above it, as the scattered light from other regions is much weaker than that in the center. Based on this observation, the top resizing location can be obtained by finding the first local maximum of intensity summation in each row by the equation $S\left(y\right)=\overset{x=X}{\underset{x=1}{\sum }}I\left(x,y\right)$ above a threshold (in this case, mean intensity summation is used) to reduce computational cost. Therefore, all rows 15 pixels above are empirically cropped. Similarly, the lower resizing position can be estimated from typical corneal thickness and the axial resolution of the image. The left and right parts are also cropped to remove unwanted structures and the low SNR region on both sides.

As shown in Figure 2, two main types of artifacts are present in OCT images: horizontal artifacts and central noise artifacts. The former appears as long horizontal stripes of high intensity, while the latter is characterized as a vertical saturation region. It is essential to eliminate these artifacts as they have a significant negative impact on the segmentation algorithm.

The horizontal artifact is characterized as adjacent rows with higher mean intensity than others, an efficient way to mitigate it is to subtract the pixel value of each row from the mean intensity of that row.

The central noise artifact is detected by finding a sudden increase in the average intensity of the A-scans, as it is characterized by relatively higher intensity pixels vertically. The whole image is divided equally into three regions and the average intensity (μ) of the A-scans in the peripheral region is calculated. Assuming that the central artifact only occurs in the central region, we therefore consider the A-scan in the central region above a certain threshold [$\frac{4}{3}\mu$ (10)] as the region contaminated by the central artifact. Once the artifact is detected, the region within the artifact is not included in the subsequent processes.

It is necessary to suppress noise in OCT images as the additive thermal and electronic noise can degrade the performance of the algorithm. A 5 × 5 Wiener denoiser (15) is used to increase the image SNR. For simplicity, the image after artifact suppression and denoising is still denoted as *I*(*x, y*).

### 2.3 Coarse segmentation of anterior surface

In this section we will focus on approximating the position of the anterior surface boundary, as the air-epithelium interface is generally the region with the best quality (high SNR) in OCT images. The main feature used in the search is the bright-to-dark or dark-to-bright transitions in the axial (vertical) direction. Instead of using a gradient with a directional filter to extract the corresponding boundary (10), a novel adapted estimation method is presented.

It is observed that relatively high pixel intensity occurs at adjacent corneal layer boundaries as a result of over-exposure of reflected and scattered light at the edge in the OCT system. Therefore, the position of the anterior interface can be easily estimated based on the prior assumption that the strongest response of an OCT system (the pixel with the highest intensity) in each A-scan mostly occurs near the corneal boundary instead of random noise. In addition, only pixels with local maxima in A-scans are considered as candidate pixels to reduce computational cost and improve accuracy by excluding other pixels.

To estimate the anterior corneal boundary, we defined a “boundary function” to characterize the corneal interface. In essence, the “boundary function” is a mathematical optimisation objective function that aims to determine the position of the boundary in each A-scan. Therefore, the anterior boundary can be obtained by maximizing the “boundary function” in a predefined region according to pixel resolution and layer thickness to find the optimal input argument. The function is defined in each A-scan in the Equation 1.


(1) $$\begin{array}{l} G\left(x\right)=T^{+}\left(x\right)-T^{-}\left(x\right) \end{array}$$

**Figure 3:**
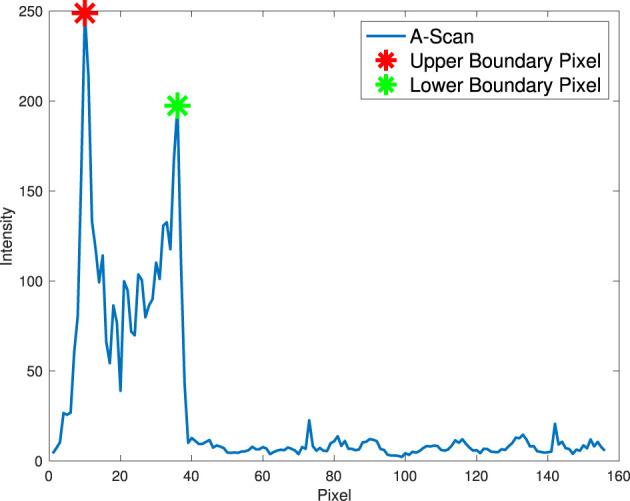
Corneal boundaries in an A-scan.

Two constraints are used in this function to characterize the corneal boundaries (Equation 2). *T*^+^(*x*) is used to account for the difference in pixel intensity between two boundary pixels as a result of over-exposure of scattered and reflected light. The second constraint, *T*^−^(*x*), aims to test the lowest intensity of all candidate pixels between two boundary pixels, as there is less scattered light within the corneal layer with similar tissue. The anterior surface is coarsely segmented by finding the top pixel in the axial direction, as shown in Figure 3.


(2) $$\begin{array}{c} T^{+}(x)=|I(x,y_{1})-I(x,y_{2})|\;y_{1},y_{2}\in [1,Y]\\ T^{-}(x)=\min_{\mu \in [y1,y2]}I(x,\mu ) \end{array}$$

Due to noise in low-SNR regions, the stability problem that some candidate pixels are in random noise should be addressed. To deal with random noise in low-SNR regions (usually in the outer parts), a second-order polynomial approximation (5, 7, 10) is used to fit the profile to eliminate the effect of random noise and form a smooth boundary (other layer boundaries without a predefined approximation model can simply use a median filter to address this problem).

### 2.4 Refinement of segmentation

In this section, a novel method is proposed to refine the boundary estimated above. In order to precisely refine the layer boundary, the tactic used is that the latter boundary pixels are determined based on the previously determined boundary pixels with a constant decay weight.

The actual boundary is defined as the maximum intensity change (dark to light or light to dark) in the axial direction. Therefore, the actual layer boundary is found by the maximum absolute vertical gradient. Assuming that the SNR in the center of the corneal image is relatively high compared to the outer part, a non-linear adjustment is considered here, which means that actual boundary pixels at the periphery need more actual central boundary pixels to be confirmed, while central boundary pixels need only a small amount of actual interface pixels to be decided. Therefore, the whole image in the center is divided into two parts to find the actual boundary.

First, the magnitude of the image gradient in the axial direction, symbolized as *g*(*x, y*), is calculated using the forward difference gradient operator $\frac{\partial I}{\partial y}=\frac{I\left(x,y+1\right)-I\left(x,y\right)}{2}$, and then for each of the latter refined boundary pixels from the center to the periphery is determined based on the maximum summation of the absolute gradient of all previously determined boundary pixels with a geometric distribution decay (constant *p*) according to Equation 3 by iteratively shifting previous refined pixels up and down in a limited search region (in this case with 5 pixels up and down from the approximated air-epithelium layer interface). The pseudocode for the detailed refinement procedure is shown in Algorithm 1.


(3) $$\begin{array}{l} y=f\left(x|p\right)=p{\left(1-p\right)}^{x};x=0,1,2,... \end{array}$$

**Algorithm 1:**
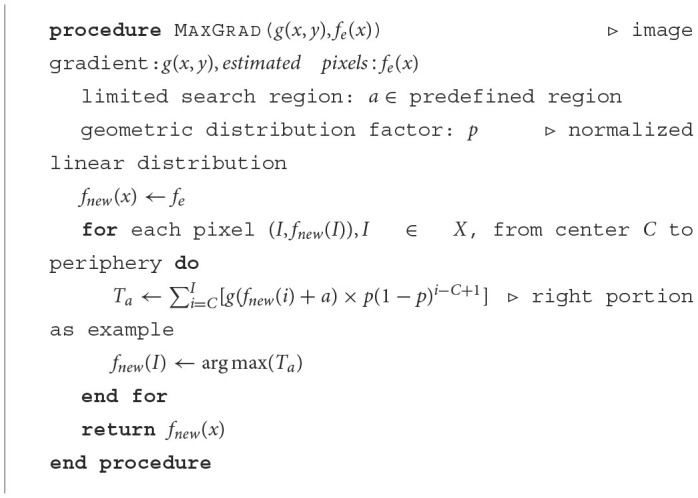
Boundary refinement.

**Figure 4:**
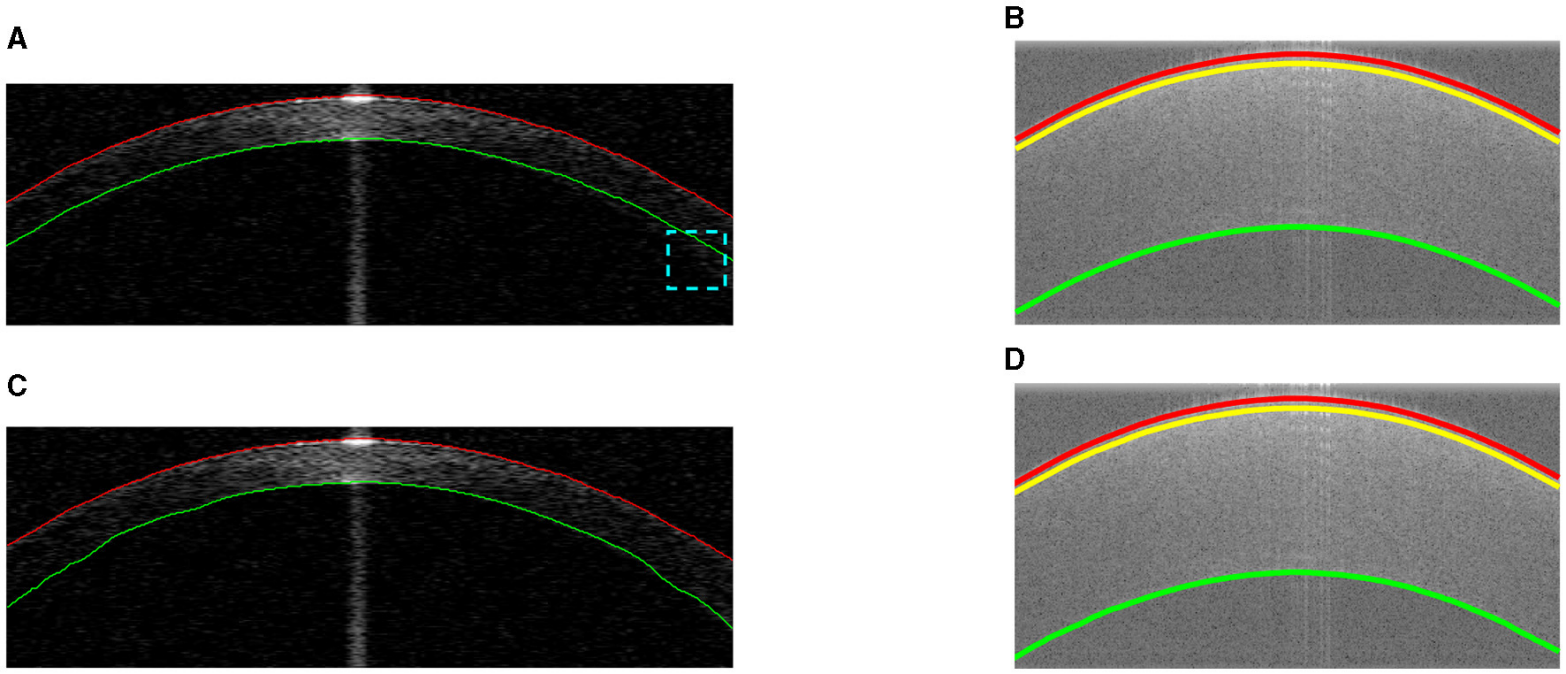
Example segmentations on different datasets. Red and green lines represent the segmented anterior and posterior layer boundaries (misalignment highlighted in cyan). The yellow line shows the epithelium-stroma interface. **(A)** Coarse segmentation in Dataset1. **(B)** Coarse segmentation in Dataset2. **(C)** Final segmentation in Dataset1. **(D)** Final segmentation in Dataset2.

After the actual air-epithelium is detected with the proposed method, to smooth the curve of the layer interface, the Savitzky-Golay filter (16–18), with the first-order polynomial and 21-frame length (11), is implemented. Figure 4 shows the refined anterior surface.

### 2.5 Estimation of other layer interfaces

Our proposed method for approximating other layers is based on the refined air-epithelium layer interface, as they will have similar boundary profiles. Other layer interfaces can be estimated by maximizing the summation of the absolute gradient, according to the Equation 4, in a region *S* vertically below the air-epithelium profile (based on typical thickness and pixel resolution). Approximations of other layers in different datasets are shown in Figure 4.


(4) $$\begin{array}{l} \arg\underset{\mu \in S}{\max}\sum g\left(x,f\left(x\right)+\mu \right) \end{array}$$

### 2.6 Refinement of other layer interfaces

Refinement of other layer interfaces based on the initial estimation in Section 2.5 is performed using a similar technique to that used to adjust the air-epithelium interface in Section 2.4. According to Liu et al. and González-Méijome et al. (19, 20), the cornea is relatively thinner at the center than at the periphery, as shown in Figure 4. Therefore, a normalized linear growth geometric distribution factor *p* (*p*∈[0, 1]) from the center to the periphery is used based on the locality of the data points, which means that the approximated data points at the periphery have more ability to explore the layer interface instead of considering the predetermined candidate pixels more than those at the center. However, due to the low SNR at the periphery near the layer boundary, the issue of segmentation stability is raised. To address this issue of refinement flexibility, the refinement technique in the Section 2.4 is again used with a low geometric distribution factor for curve smoothing. Figure 4 shows the final segmentation of all visual corneal layers.

### 2.7 Three-dimensional segmentation

Reconstruction of 3D surface maps of the cornea follows the method for segmentation of 2D B-scans described in the previous sections. There are three main steps: preprocessing, estimation and refinement.

Similar processing steps are implemented to crop the 3D volume image to ensure that only the ROI remains and that various artifacts are removed.

To estimate the air-epithelial interface, the “boundary function” approximation described in Section 2.3 is used with a quadratic surface fit to eliminate the effect of random noise. A similar refinement technique is used in Section 2.4. In 3D segmentation, the starting pixel is in the center of the cornea as we assume that high resolution is presented in the center of the image while low SNR appears in the periphery. For non-linear adaptation, additional information from neighboring pixels was introduced. Candidate pixels are considered using geodesic distance transform (here the city block method is used) (21). Each candidate pixel from the center to the periphery (the distance after transformation) is determined based on the maximum summation of the absolute gradient of all previously refined pixels with geometric distribution decay in a limited region, which means that the algorithm considers more the neighboring candidate pixels and less the distant candidate pixels. A 3 × 3 × 3 median filter is used for curve smoothing.

Other layer interfaces can be approximated by maximizing the sum of the absolute gradient of all estimated pixels in a limited region by shifting the refined air-epithelium profile, following Equation 5.


(5) $$\begin{array}{l} \arg\underset{\mu \in S}{\max}\sum g\left(x,y,f\left(x,y\right)+\mu \right) \end{array}$$

**Figure 5:**
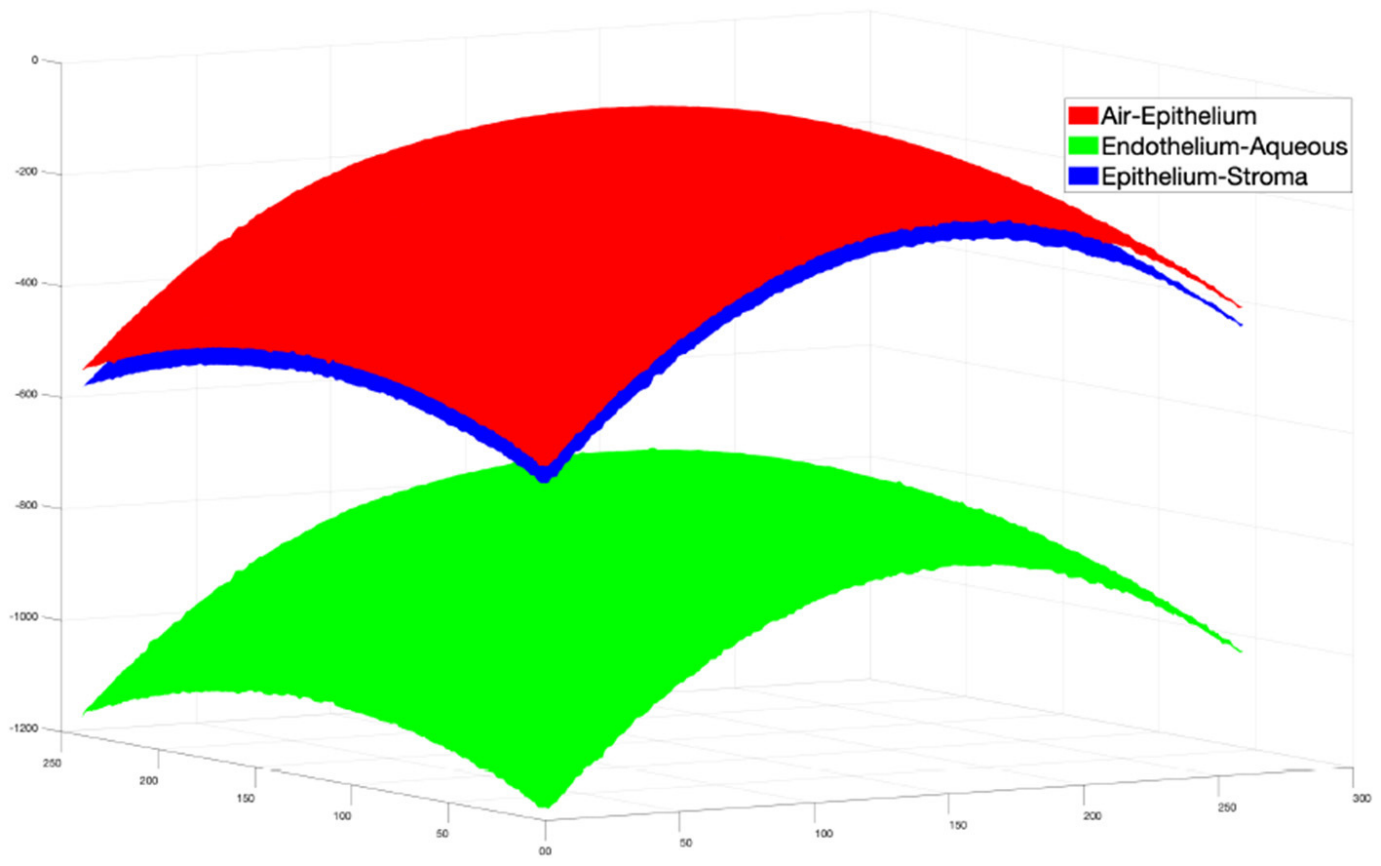
Surfaces reconstructed from 3D segmentation.

Then the same refinement method mentioned above is used to find the actual boundary pixels. The final segmentation result is shown in Figure 5.

### 2.8 Experiments

The algorithm is implemented in Matlab and runs without parallel processing on a Win10 64-bit OS PC with Intel Core i5-7500 CPU @ 3.40 GHz and 8.00 GB RAM.

To evaluate the performance of our method and the other (13), the segmentation results of different layer surfaces were compared with the ground truth described in Section 2.1. The mean unsigned surface position error (MSPE) (13) is used as a metric to evaluate performance. In order to examine the intra- and inter-observer agreement, for Dataset1 the annotations of the same observers and between observers were also compared. For Dataset2, due to data availability, only intra-observer variation was assessed.

## 3 Results and discussion

The proposed method was first compared with Williams et al. (13), which used dataset1. The comparison results between the proposed method and that of Williams et al. are summarized in Table 1. A significant improvement in accuracy can be observed: our mean ± standard deviation MSPEs on the anterior and posterior interfaces are 0.62 ± 0.61 and 2.15 ± 2.26 pixels, while theirs are 1.21 ± 1.64 and 2.82 ± 1.26 pixels. In addition, the processing time of our proposed algorithm (0.74 *s*) is much lower than theirs (2.53 *s*). Furthermore, an observer variation test was also performed, as shown in Table 1. The mean pixel error of our method for the anterior boundary (0.62 ± 0.61) is lower than the interobserver variation (0.80 ± 0.90) and the intraobserver variation (0.92 ± 1. 46), and the mean pixel error of our method for the posterior boundary (2.15 ± 2.26) is slightly higher than the inter-observer variation (1.25 ± 1.46) and the intra-observer variation (1.62 ± 2.33). These results demonstrate the good performance of our proposed method.

**Table 1:** Results on Dataset1 in mean unsigned surface positioning error.

**Corneal layer boundary**	**Proposed method**	**Williams' method**	**Inter-observer variation**	**Intra-observer variation**
Anterior	0.62 ± 0.61	1.21 ± 1.64	0.80 ± 0.90	0.92 ± 1.46
Posterior	2.15 ± 2.26	2.82 ± 1.26	1.25 ± 1.46	1.62 ± 2.33

The proposed method was also tested on Dataset2 and the results are summarized in Table 2. The MSPE in pixels for all three layers (2.67 ± 0.40 pixels for epithelium-air, 2.30 ± 0.40 pixels for epithelium-stroma and 2.62 ± 0. 44 pixels for endothelium-aqueous) are lower than those of the intra-observer variation (4.47 ± 5.76 pixels for epithelium-air, 5.90 ± 4.25 pixels for epithelium-stroma, and 5.14 ± 3.13 pixels for endothelium-aqueous). Considering the high resolution of the images, the actual error is comparatively small, e.g. the MSPE is 5.2 μ*m* for the endothelium-aqueous layer interface, which is comparable to those of other methods (10, 11, 13). The technique has demonstrated high speed segmentation with an average segmentation time of 0.42 seconds per image for three visible interfaces.

**Table 2:** Results on Dataset2 in mean unsigned surface positioning error.

**Corneal layer boundary**	**Mean ± standard deviation**	**Intra-observer variation**
Epithelium-air	2.67 ± 0.40	4.47 ± 5.76
Epithelium-stroma	2.30 ± 0.39	5.90 ± 4.25
Endothelium-aqueous	2.62 ± 0.44	5.14 ± 3.13

To test the performance of our extended 3D segmentation method, the surfaces of the layer interfaces were constructed using our proposed 2D segmentation method on each B-scan image in Dataset2. In our experiment, B-scans from 51 to 310 were segmented to construct the surfaces. The surfaces from the direct 3D segmentation method were compared with those from the 2D constructed surfaces, and the results are shown in Table 3.

**Table 3:** 3D segmentation results in Dataset2.

**Mean unsigned surface positioning error (MSPE)**	
**Corneal layer boundary**	**Mean** ± **standard deviation**
Epithelium-air	7.06 ± 9.03
Epithelium-stroma	7.03 ± 10.31
Endothelium-aqueous	12.47 ± 13.10

**Figure 6:**
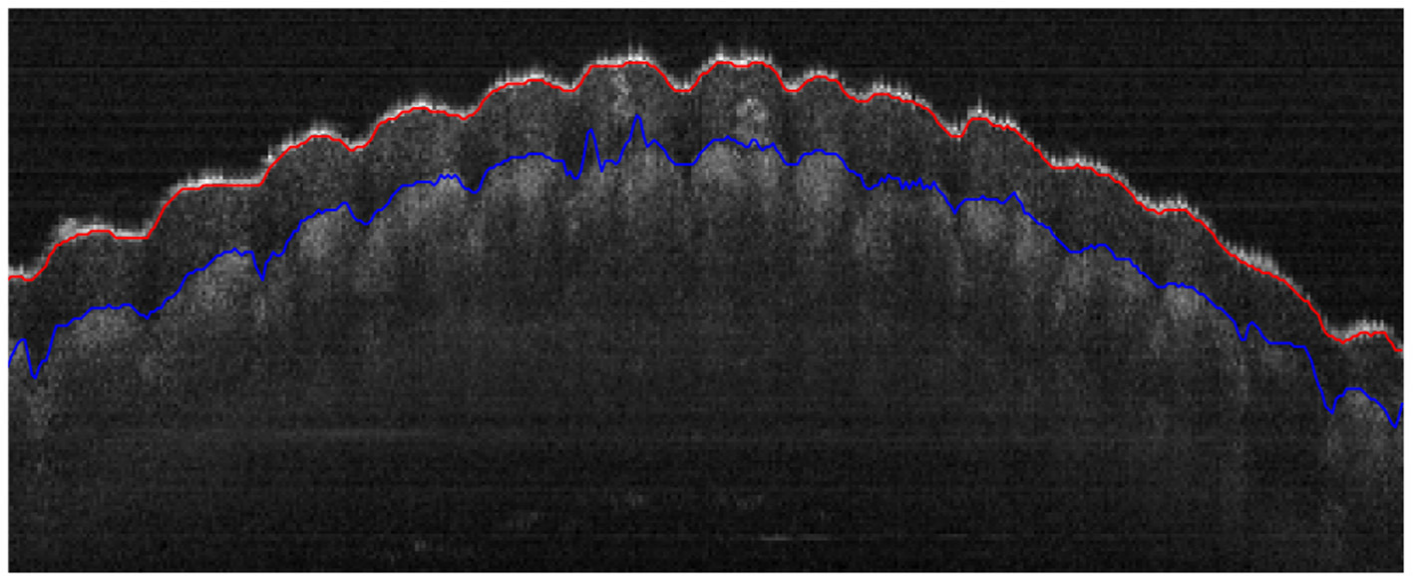
Segmentation of the epidermal layer of a fingerprint with uneven surfaces, where the red and blue curves represent the anterior and posterior layer interfaces, respectively.

To further demonstrate the robustness of the proposed algorithm, an OCT image of a fingerprint image was segmented and the results are shown in Figure 6. Although the layer interfaces are more irregular compared to those of the cornea, the results are appealing and demonstrate the robustness of our method in dealing with complex surfaces such as the diseased cornea in the future.

## 4 Conclusion

A novel technique for automatic segmentation of corneal layer interfaces in OCT images has been proposed and validated. The proposed method outperforms state-of-the-art methods in terms of accuracy and time efficiency. The method is extended to 3D segmentation with relatively high accuracy. The method could be used to segment more layer boundaries resolved by OCT imaging techniques. Thus, the method has significant potential for clinical care.

## Data availability statement

The raw data supporting the conclusions of this article will be made available by the authors, without undue reservation.

## Ethics statement

The studies involving humans were approved by the Ethics Committee of Cixi Institute of Biomedical Engineering, Chinese Academy of Sciences. The studies were conducted in accordance with the local legislation and institutional requirements. The participants provided their written informed consent to participate in this study.

## Author contributions

XZ: Data curation, Formal analysis, Investigation, Methodology, Validation, Visualization, Writing – original draft, Writing – review & editing. WH: Methodology, Project administration, Resources, Writing – original draft, Writing – review & editing. SM: Conceptualization, Funding acquisition, Investigation, Project administration, Software, Visualization, Writing – review & editing. QY: Data curation, Formal analysis, Funding acquisition, Investigation, Methodology, Supervision, Validation, Writing – review & editing.
